# Preventative Approach to Microbial Control of *Capnodis tenebrionis* by Soil Application of *Metarhizium brunneum* and *Beauveria bassiana*

**DOI:** 10.3390/insects11050319

**Published:** 2020-05-22

**Authors:** Dana Ment, Hysen Kokiçi, Enrico de Lillo

**Affiliations:** 1 Department of Entomology, Nematology and Chemistry Units, Agricultural Research Organization, Volcani Center, Rishon LeZion 7505101, Israel; 2 Dipartimento di Scienze del Suolo, della Pianta e degli Alimenti (DiSSPA), Sezione di Entomologia e Zoologia University of Bari “Aldo Moro”, I-70126 Bari, Italy; hysenkokici1989@gmail.com (H.K.); enrico.delillo@uniba.it (E.d.L.)

**Keywords:** Mediterranean flat-headed root-borer, microbiological control, entomopathogenic fungi, fungal efficacy, soil application, fungal survival

## Abstract

Management of the Mediterranean flat-headed root-borer, *Capnodis tenebrionis*, is critical due to the larvae’s root localization. Neonate larvae can be exposed to natural enemies before penetrating the roots. Application of *Metarhizium brunneum* strain Mb7 and *Beauveria bassiana* strain GHA formulations on rice granules was investigated for their efficacy against *C. tenebrionis* larvae. Mb7 application, evaluated on apricot twigs, significantly and dose-dependently reduced colonization rates of neonates, with highest mortality at 10^8^ conidia/g soil. Neonate susceptibility to Mb7 and GHA was evaluated on potted rootstocks (GF677 almond × peach, 2729 plum) planted in entomopathogenic fungi (EPF)-premixed soil (1.3–1.6 × 10^5^ conidia/cm^3^ soil) or in EPF-free soil surface-treated with 5 g Mb7 fungal granules (1.25 × 10^9^ conidia). Larval colonization rates were reduced 7.4-fold in 2729 by both fungi; only Mb7 completely prevented colonization of GF677 by larvae. Larvae inside plant galleries exhibited mycosis with EPF-treated soils and both fungi proliferated on larval frass. Mb7 conidia germinated in the rhizosphere of GF677, and conidia of both fungi remained viable throughout the trial. *Galleria* baiting technique was used on EPF-treated soil to evaluate EPF infectivity over time; Mb7 and GHA persisted 180 and 90 days post inoculation, respectively. The formulation (fungus-covered rice grains), delivery method (mixing with soil) and persistence (3–6 months) of Mb7 and GHA are feasible for potential field application to control *C. tenebrionis*.

## 1. Introduction

The Mediterranean flat-headed root-borer, *Capnodis tenebrionis* (L.) (Coleoptera: Buprestidae), can severely damage ornamental and stone-fruit tree species, and is a key pest of apricot, peach, plum, nectarine, cherry, and almond, especially under arid and semiarid conditions with scarce water supply, and in organic orchards [1,2]. The life cycle of *C. tenebrionis* is characterized by the tree-canopy phase of the adults, which feed on the bark of shoots, buds, and leaf petioles causing defoliation, and by the endophytic root phase of the juveniles, in which all larval instars feed on the root cortical and subcortical tissues causing deficiencies in the plant’s vascular system [3,4,5,6]. Under natural conditions, the eggs are laid during summer—with some slight variations in the involved months based on the thermal profiles of the different climatic areas in the Mediterranean Basin [7,8,9,10]. The eggs are glued among soil particles on the superficial ground layer, mainly close to the trees [11]. Soon after hatching, neonate larvae crawl through the soil toward the host plant roots and penetrate them [12]. During this short searching time, the neonate larvae are exposed and vulnerable to natural enemies inhabiting the soil [7,12]. This pest is mainly controlled at the adult stage by repeated foliar sprays of chemicals. The difficulties in establishing the right application timing and the poor availability of chemicals against *C. tenebrionis* have encouraged the search for alternative management strategies. These are particularly relevant in the context of organic cultivation and with the recent restrictions imposed by the European Union directives on plant-protection products [13].

Research in recent years has addressed the efficiency of *C. tenebrionis* control by entomopathogenic fungi (EPF) and nematodes [12,14,15,16,17,18,19,20,21,22,23], showing their high impact on the soil-crawling neonates. Almost all investigations on EPF applications against *C. tenebrionis* have evaluated the efficiency of the EPF against eggs, larvae, and adults, with larvae appearing to be the main target. Larvae have been successfully infected with isolates of *Beauveria bassiana* (Balsamo) Vuill. and *Metarhizium anisopliae* (Metchnikoff) Sorokin by dipping them into a conidial suspension [16] or by applying conidial suspensions to soil [17]. Those results indicated that both EPF are promising candidates for studying prevention of larval infestations of roots. However, the EPF’s persistence in the soil and their mode/timing of application were not detailed enough. Considering the recent revision of the *Metarhizium* species complex [24], more accurate data concerning one of its species, *Metarhizium brunneum* Petch., are also needed, because variations in virulence and efficacy have been previously recognized when applied against other target species [25,26].

The current work studied the susceptibility of *C. tenebrionis* neonate larvae to *M. brunneum* strain Mb7 and the commercial strain GHA of *B. bassiana* under laboratory and semiartificial conditions, with the purposes of evaluating (i) the efficacy of different EPF-application modes, (ii) the survival of the fungal conidia, (iii) the persistence of their efficacy in the experimental system.

## 2. Materials and Methods

### 2.1. Fungal Strains and Their Mass Production

Isolates of *M. brunneum* strain Mb7, previously referred to as *M. anisopliae*-7 [26], and *B. bassiana* strain GHA (commercial product BotaniGard^®^ ES, LAM International, Butte, MT, USA) were used. They were transformed to constitutively express green fluorescent protein (GFP) [26]. Mb7-GFP and GHA-GFP were grown on Sabouraud dextrose agar for 2 weeks at 28 ± 0.5 °C. Conidia were harvested by scraping the agar and suspending them in sterile distilled water containing 0.01% (v/v) Triton X-100 in glass tubes. The suspension was vortexed and filtered through Miracloth (Calbiochem, La Jolla, CA, USA). The conidial concentration was determined with a hemocytometer and adjusted to 10^8^ conidia/mL; 100 μL of this suspension was transferred to a 250 mL Erlenmeyer flask with 100 mL sterile Sabouraud dextrose broth containing 100 μg/mL chloramphenicol. This mixture was incubated for 3 days on a shaker at 150 RPM and 28 ± 0.1 °C. Blastospore production was verified by observing a sample of the mixture under a microscope. Organic rice (*Oryza sativa* L.) was soaked in distilled water for 30 min and left to dry outdoors until it became firmer and less sticky; 500 g of rice grains was transferred into a fermentation spawn bag (SacO_2_, Belgium; cat. No. PP75-BEH4+1-V22-49) and autoclaved. After cooling, 35 mL of the liquid fermentation of one of the EPF was aseptically poured into each bag. Bags were carefully shaken, sealed and left in an incubator in the dark at 28 ± 1 °C for Mb7-GFP and at 25 ± 1 °C for GHA-GFP, to allow EPF sporulation which occurred after 7–10 days of incubation. These grains of rice, hereafter termed fungal granules, were used immediately after production. Each gram of fungal granules contained 2.5 × 10^8^ Mb7-GFP conidia or 1.5 × 10^8^ GHA-GFP conidia.

### 2.2. Stock Breeding of C. tenebrionis

Adult beetles were collected from infested apricot orchards in the Upper Galilee district (Israel) from June to September 2018 and 2019, by hand or using an entomological umbrella. Active, healthy adults were held in netted, wooden cages (50 × 30 × 30 cm). Beetles were maintained in a screen house at 27 ± 1 °C and fed on fresh apricot and peach twigs. Cages were inspected every 5–7 days to replace old twigs with fresh ones, and remove feces and dead individuals. Adults were left to mate and lay eggs in the same cage, provided with 6–8 soil arenas [27]. Eggs were incubated at 27 ± 1 °C for larval hatching.

### 2.3. Susceptibility of C. tenebrionis Neonates to Mb7-GFP under Laboratory Conditions

**Figure 1:**
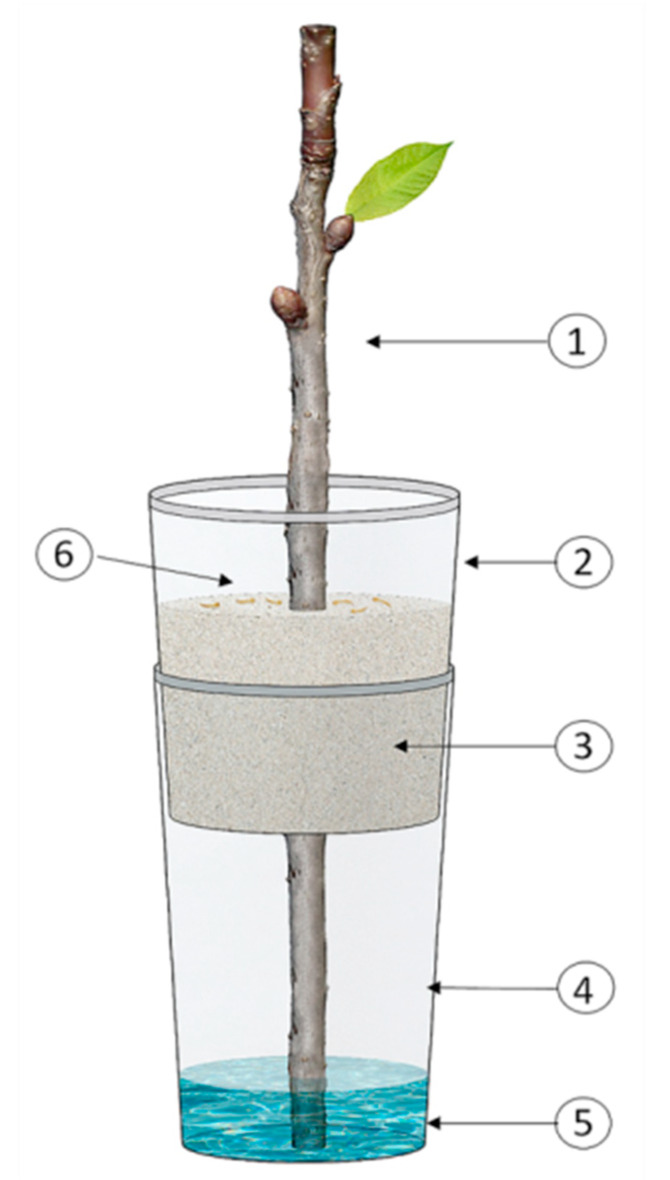
Schematic of the experimental microcosm applied to evaluate the susceptibility of *Capnodis tenebrionis* neonates to Mb7-GFP under laboratory conditions modified from Azoulay [28]: (1) Apricot twig; (2) 250 mL cup; (3) Sandy soil mixed with Mb7-GFP or fungus-free (control); (4) 500 mL cup; (5) 2.5 cm layer of water; (6) Site of *C. tenebrionis* neonate release.

The susceptibility of *C. tenebrionis* neonates to the Mb7-GFP strain was assessed under laboratory conditions following the experimental model by Azoulay [28] (Table 1). Even though twigs are not the elective organs preferred by larvae, they are simple to manage in the microcosms and they keep their attractiveness towards the larvae which can develop easily into them [3,16,28]. The following microcosm was designed to carry out the assay (Figure 1). The basal part of an apricot twig of about 0.9 cm diameter was inserted in a hole made at the bottom of a 250 mL cup (1 cm diameter). The upper twig’s cut edge was sealed with Parafilm-M to prevent water evaporation and desiccation. The leaves were stripped off. New leaves started to emerge during the experiment. This first cup was pushed into a bigger cup (500 mL) with the same diameter, containing water on the bottom (to about 2.5 cm of the cup height) in which the twig base was dipped. About 450 g of dry and sterilized (at 121 °C for 15 min) sandy soil without (control) and with Mb7-GFP was added to the small cup, surrounding the twig. In the Mb7-GFP treatment, 50 g of soil was mixed with fungal granules and placed over the sandy soil previously used. In the control microcosms, 50 g of EPF-free soil was placed over the sandy soil previously used. Then, 10–15 neonates (no more than 24 h old) per microcosm were transferred onto the topsoil near the twig. Microcosms were incubated at 25 ± 1 °C for 14 days.

A first trial was carried out with a twig group treated with 5 × 10^6^ conidia/g soil and its control (EPF-free soil). A second trial was performed on groups of twigs treated with 10^5^, 10^6^, and 10^8^ conidia/g soil, and its control (EPF-free soil) to evaluate the optimal application dose. Five replicates were carried out for each treatment. The bioassays were repeated four times.

The infestation and colonization rates of twigs and mycosis on *C. tenebrionis* larval cadavers were assessed 14 days after releasing neonates to the microcosm. Soil was inspected to detect dead larvae and twigs were peeled to expose larvae and their galleries. The infestation rate was calculated as percentage of twigs with boring signs out of total number of twigs used per treatment. The colonization rate was expressed as percentage of larvae found in the infested twigs out of the total number of larvae released per treatment. Mycosis was assessed by looking for developing fungi on the surfaces of larvae found in the soil and twigs. Dead larvae without detectable signs of mycosis were incubated under moist conditions until the fungi developed. The presence of Mb7-GFP in dead larvae was confirmed by confocal laser scanning microscopy (CLSM) (Leica SP8/LAS X). Mycosis rate was calculated as percentage of cadavers with Mb7-GFP mycosis out of total number of cadavers.

### 2.4. Preparation of the Fungal Premix Soil

Non sterilized soil was used to pot rootstocks. This soil consisted of 50% red soil, 25% sand, and 25% stones. Fungal granules of Mb7-GFP or GHA-GFP were added to this soil. Mb7-GFP was incorporated at a rate of 3 g (7.5 × 10^8^ conidia) fungal granules/L potting soil in the 2018 and 2019 trials. GHA-GFP was incorporated at a rate of 4 g (6 × 10^8^ conidia) fungal granules/L potting soil in the 2019 trial. The doses were approximately to those applied in previous studies [17,29].

**Figure 2:**
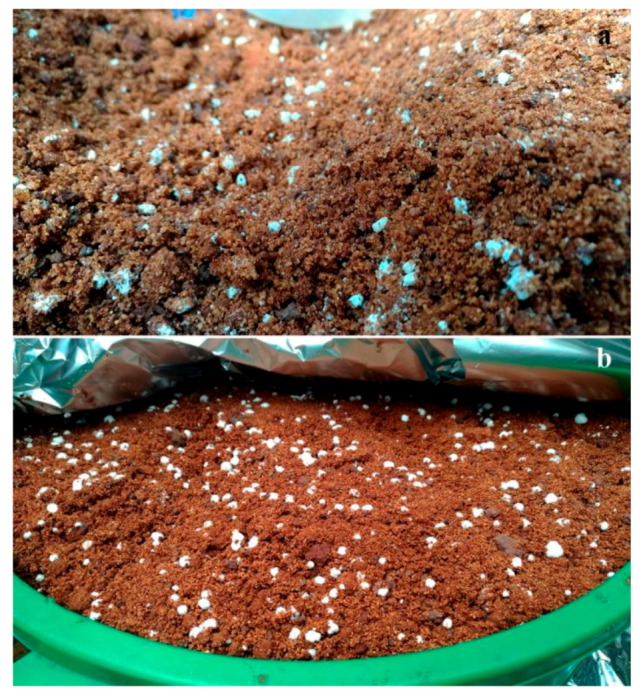
Fungi developed in the premix soil after 7 days of incubation: (**a**) Mb7-GFP; (**b**) GHA-GFP.

Fungal premix soil was prepared similarly to a previous study [29] by mixing the ingredients for 7 min to ensure uniform incorporation of the conidia. Before planting in it, premix soil was allowed to incubate for 7 days at 26 ± 2 °C in pails covered with aluminum foil and left in the greenhouse head house. Hyphal growth was evident when the foil cover was removed (Figure 2).

### 2.5. Susceptibility of C. tenebrionis Neonates to Mb7-GFP and GHA-GFP Strains on Potted Rootstocks

Rootstock-colonization rate by *C. tenebrionis* larvae and larval mycosis were examined for potted plants in three treatments: (i) Mb7-GFP (2018 and 2019 trials) and GHA-GFP (2019 trial) premix soil; (ii) distribution of 5 g of Mb7-GFP fungal granules on the top of EPF-free soil around the rootstock (topsoil application) (2018 trial); (iii) untreated soil (control) (2018 and 2019 trials) (Table 1).

In the 2018 trial, two-year-old rootstocks of GF677 (*Prunus persica* (L.) Batsch × *P. dulcis* Webb, i.e., almond × peach) and 2729 (*P. domestica* L., plum) were potted with a volume of 0.00458 m^3^ premix soil and were maintained in a screen house for 5 months before larval release. This procedure was performed to give Mb7-GFP, premixed in the soil, and given the chance to germinate and grow, thus potentially increasing the overall inoculum level. Other plants of the same rootstocks were planted in the same amount of EPF-free soil and with the same storage method/time as above. A day before the release of the larvae, Mb7-GFP fungal granules were spread on the soil surface of half of the rootstocks planted in EPF-free soil. The remaining planted rootstocks were not treated and were used as control. Each treatment consisted of three to four plants of each rootstock (two groups of four plants as untreated) (Table 1).

In the 2019 trial, two-year-old rootstocks of GF677 and 2729 were also used. They were potted in the same premix soil or EPF-free soil volume and maintained in a screen house for 4.5 months before larval release. Only Mb7-GFP and GHA-GFP premix soils were used. Each treatment consisted of six to eight plants (two groups of eight plants as untreated) (Table 1).

For both 2018 and 2019 trials, the potted rootstocks were kept without watering for 5 days before releasing 20 neonate larvae per each plant. Neonate larvae (no more than 24 h old) were placed on the topsoil at a distance of 1–7 cm from the rootstock base. Rootstocks were kept without watering for 3 days after larval release to make the root more accessible and better suited to neonate colonization.

Two months after neonate release in both 2018 and 2019 trials, the soil was gently removed from the rootstock roots. They were carefully examined for any signs left by larvae while boring (gum secretion and erosion) and, then, peeled to detect live or dead larvae. The infestation and gumming rates were calculated as percentage of plants with boring or gumming signs, respectively, out of the total number of plants assayed for each treatment. In the 2018 trial, the plant-colonization rate by *C. tenebrionis* larvae was expressed as percentage of plants infested by at least one live larva per treatment out of total number of plants assayed for each treatment. The results were binomial and, due to low number of replicates, statistical analysis was not done. In the 2019 trial, the larval colonization rate of the plants was expressed as percentage of number of larvae found in the infested rootstock out of the total number of neonates released per pot. At the end of the 2019 trial, the mycosis rate was calculated as a percentage of larvae with mycosis out of the total number of larvae released to each plant. The length of the galleries excavated by the larvae was also measured.

### 2.6. Survival and Infectivity of the Fungus over Time in Potted Rootstocks

Fungal survival and development in the soil and rhizosphere were monitored over time in 2018 and 2019 from the same potted rootstocks used for studying the susceptibility of neonates to premix soil treatment. Soil and root samples were collected from each pot at random spots at 0, 14, and 42 days after planting for 2018, and at 0, 90, and 180 days after planting for 2019.

Three (2018) and five (2019) 50 g soil samples were taken, using a shovel, from the topsoil and inside the rhizosphere of each pot, each treatment and at each time. These samples were analyzed separately. Soil subsamples of 2–3 g per sample were placed in a 1.5 mL sterile Eppendorf tube with distilled water and 0.01% Tween-80, and the tube was vortexed for 20 s. A 10 µL aliquot of the suspension was pipetted onto a glass slide and immediately observed by CLSM (Leica SP8/LAS X). GFP was excited at 488 nm wavelength with an argon laser and visualized at 495–550 nm wavelengths.

Root samples were selected randomly, cut to a size of 2–5 cm in length and 1 mm in diameter with a sterile scalpel, placed on a glass slide, and observed by CLSM using the same excitation wavelengths as above.

The infectivity of the premix fungi over time was investigated by means of *Galleria mellonella* L. larvae [30] on the soil samples collected above. Soil samples were taken also from untreated soil. Soil from each sample was placed into five polystyrene, non-vented Petri dishes (90 mm diameter). Five fifth-instar larvae of *G. mellonella* obtained from a laboratory colony held at 23 °C at the Volcani Center [31] were placed on the surface of each dish. The dishes were sealed with Parafilm and incubated at 28 ± 0.5 °C for 7 days. They were turned over daily to ensure and facilitate larval movement through the soil. After 7 days of incubation, the soil was examined for dead larvae, which were removed. If dead larvae did not exhibit mycosis at the inspection, they were surface sterilized in 1% sodium hypochlorite for 30 s and washed three times in fresh sterile distilled water. These larvae were placed on sterile wet filter paper in sterile, non-vented, polystyrene Petri dishes, which were sealed with Parafilm, incubated at 28 ± 0.5 °C, and inspected daily for the presence of fungal mycelium and sporulation. Mycosis rate was calculated as percentage of *Galleria* larvae showing mycosis out of total number of *Galleria* larvae in the Petri dish.

### 2.7. Statistical Analysis

Datasets were analyzed using the SAS software program (SAS Institute, 2003). Colonization and infestation rates were analyzed after arcsine transformation. Treatments with an average percentage of 0 or 100 were not included in the statistical analysis. When the overall analysis of variance (ANOVA) F statistic for the treatments was significant (*p* < 0.05), they were compared by Tukey test (*p* < 0.05). The same procedure was applied for gallery length data. The effect of treatment and rootstock on signs of boring and gumming was analyzed by nominal logistic regression. Rate of *G. mellonella* larvae with mycosis 7 days after inoculation were arcsine-square-root transformed and subjected to ANOVA. If differences among treatment means were found to be significant (*p* < 0.05), Tukey’s test (*p* < 0.05) was used for multiple comparisons among means for the treatments and time post-inoculation. When one or two treatments showed zero variation, 95% confidence limits were calculated for the means of the remaining treatments. Two treatments with variations were compared by Student’s *t*-test. A significant difference was declared from 0 or from 100 if the transformed value of 0 or 100, respectively, was not included in the confidence interval. For the *t*-test and confidence limits, the significance and confidence levels were set at 0.05/3 = 0.017 in accordance with the Bonferroni correction.

## 3. Results

### 3.1. Susceptibility of C. tenebrionis Neonates to Mb7-GFP under Laboratory Conditions

The infestation rate of twigs in the control microcosms did not differ from that of twigs in the microcosms treated with 5 × 10^6^ Mb7-GFP conidia/g soil (100% and 78.0 ± 14.1%, respectively) (Table 2). However, the colonization rates of the two groups differed significantly in microcosms and they were 86.0 ± 14.0% for the control and 20 ± 5.7% for the group treated with 5 × 10^6^ conidia of Mb7-GFP per g of soil (Table 2). Neonates were not detected on the soil surface in either treatment. Mycosis was not observed on larvae recovered from control twigs, but was observed on larvae recovered from the Mb7-GFP treatment (60.0 ± 22.4%) (Table 2).

A dose-dependent effect was observed in the microcosms treated with increasing doses of conidia on the infestation rates of twigs; 100% for the control group, 76 ± 7.5 and 48 ± 4.9% for 10^5^ and 10^6^ conidia of Mb7-GFP per g of soil, respectively (Table 3). No infestation signs were observed in the twigs at 10^8^ conidia/g soil. With respect to colonization rates, no larvae were found in the twigs at 10^8^ conidia/g soil, indicating a significant difference from the control (F = 443.8, DF = 1, *p* < 0.0001; Table 3). Significant differences were detected in colonization rates between the control and the three assayed fungal doses (DF = 3, F = 31.019, *p* < 0.0001; Table 3). No difference was detected in the rate of dead neonates on the soil surface between the control group and the three treatment doses (F = 0.044, DF = 2, *p* = 0.9571; Table 3).

**Figure 3:**
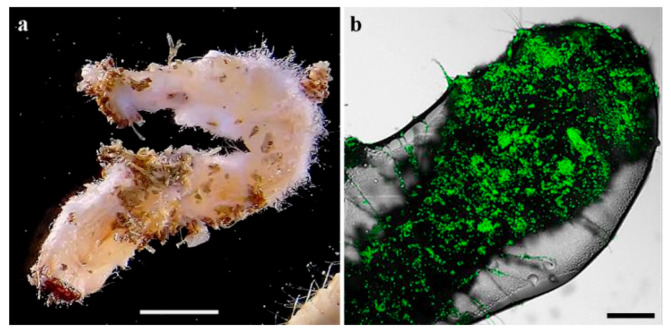
*C. tenebrionis* larvae infected by Mb7-GFP, 14 days after larval release in the microcosms: (**a**) larva found in a twig; (**b**) larva covered with conidia. Scale bar = 5 mm (**a**) and 0.2 mm (**b**).

Larvae exhibiting mycosis were visible on the soil surface, as well as in the twigs (Figure 3a). Dead larvae without evident signs of fungal infection during the inspection were incubated under moist conditions and developed mycosis. GFP-expressing Mb7 was confirmed by CLSM on cadavers (Figure 3b). No signs of mycosis were observed in larvae from the control group.

### 3.2. Susceptibility of C. tenebrionis Neonates to Mb7-GFP Strain on Potted Rootstocks in 2018 Trial

The 2018 trial compared the effects of Mb7-GFP by premix soil application to those by topsoil application. The results had only preliminary relevance in addressing the most efficient mode of application for the 2019 trial, because of the few replicates got at the end of the trial (many potted plants did not survive before larval release). Neonates were observed moving toward the rootstocks and penetrating the soil within 5–10 min after their release in both assayed modes of fungal application.

The infestation and colonization rates of the plants for the control group, and the Mb7-GFP premix and topsoil applications are given in Table 4. All infested plants showed heavy damage by the larvae, represented by large signs of boring, some of them only on the roots and some others also extending into the base and other parts of the young trunk. Larvae found in treated and untreated plants were healthy and did not exhibit any sign of mycosis on their body. In the plants potted in EPF-free soil, a higher rate of gumming was observed than for either Mb7-GFP application mode (Table 4).

### 3.3. Susceptibility of C. tenebrionis Neonates to Mb7-GFP and GHA-GFP Strains on Potted Rootstocks in 2019 Trial

Based on the preliminary data obtained in 2018, fungal granules of either Mb7-GFP or GHA-GFP were included in the soil premix and assayed for control of the beetle neonate larvae.

**Figure 4:**
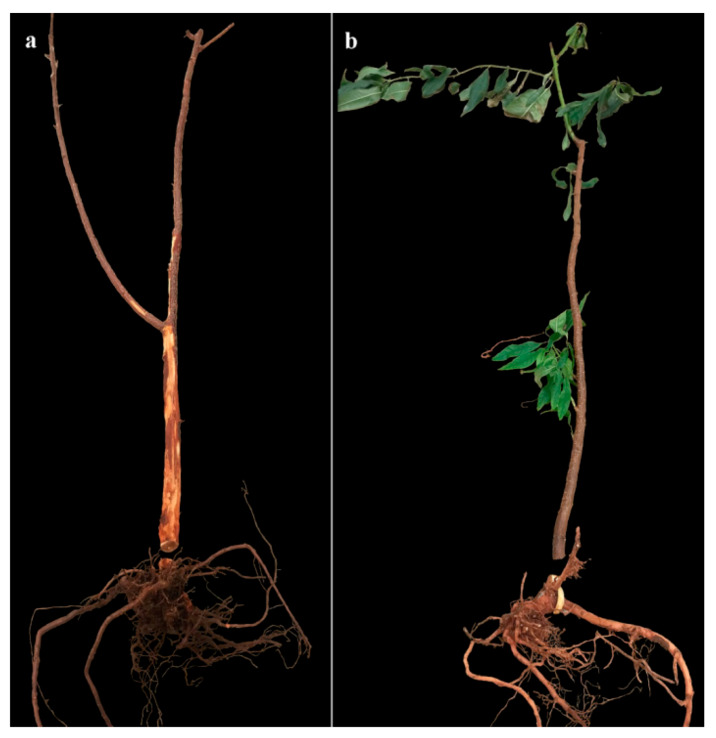
Inspected 2729 (**a**) and GF677 (**b**) rootstocks from control treatment at the end of the 2019 trial, showing their root system; leaf drop and galleries extending into the young trunk are visible in (**a**).

Before inspection, leaves of all untreated 2729 and 25% of untreated GF677 plants dropped off or withered (Figure 4). The infestation rates of control group rootstocks were highly separated from those on rootstocks of both EPF treatment groups (Treatment: Chi-Square = 9, DF = 2, *p* = 0.011. Rootstock: Chi-Square = 4.5, DF = 1, *p* = 0.0338; Table 5, Figure 4). The larval colonization rate was higher in 2729 than in GF677 control rootstocks, as well as relative to all treatments (Table 5). Significant differences in larval colonization rates were observed between 2729 control rootstocks and fungal treatments (F = 25.1, DF = 2, *p* < 0.0001). Significant differences in larval colonization rates were observed between GF677 control plants and Mb7-GFP-treated plants (*t*-test DF = 7, *p* = 0.0021) (Table 5). Rates of dead larvae inside both types of rootstocks ranged from 0% to 3.3% with no differences among treatments (Table 5). Significant differences in mycosis rate and gallery length were observed between control and treated 2729 rootstocks (F = 5.21, DF = 2, *p* = 0.0172; F = 11.7, DF = 2, *p* = 0.0024; respectively) (Table 5).

**Figure 5:**
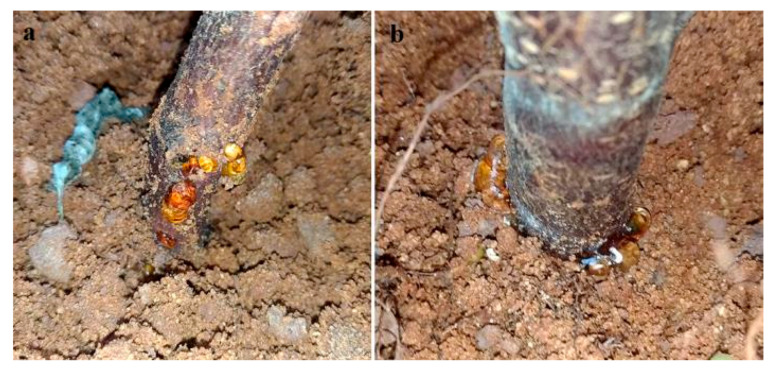
Gumming at the rootstock base 15 days after larval release: (**a**) detail of the trunk base without adhering soil; (**b**) detail of the trunk base and gum secretion at the level of the ground.

Higher gumming rates were observed from the untreated plants of both rootstocks in comparison to both treatments (Chi-Square = 12.78, DF = 2, *p* = 0.0017; Table 5). Gumming started 15–20 days after the larvae release and occurred on the young trunk near the soil (Figure 5). In the control groups, gumming appeared for all 2729 plants and only for 62.5% of the GF677 plants (Chi-Square = 2.5, DF = 1, *p* = 0.1).

**Figure 6:**
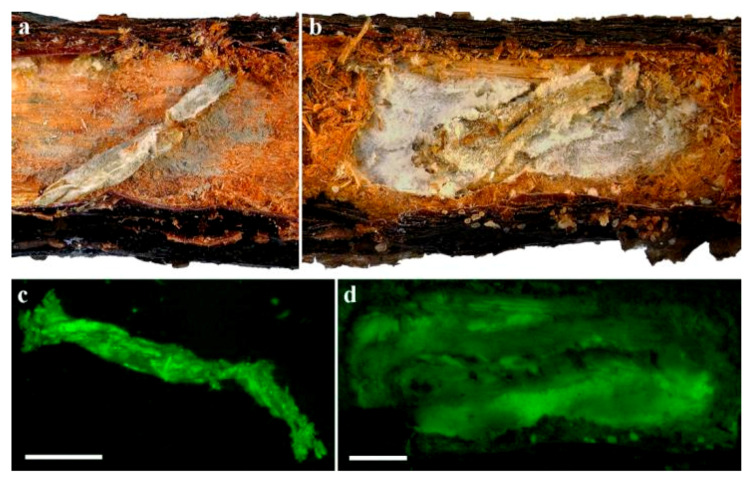
*C. tenebrionis* larvae with Mb7-GFP mycosis on 2729 rootstocks (**a**,**c**) and GHA-GFP mycosis on GF677 rootstocks (**b**,**d**). Micrographs (**c**) and (**d**) were taken under a fluorescent stereomicroscope. Scale bar = 2 mm.

**Figure 7:**
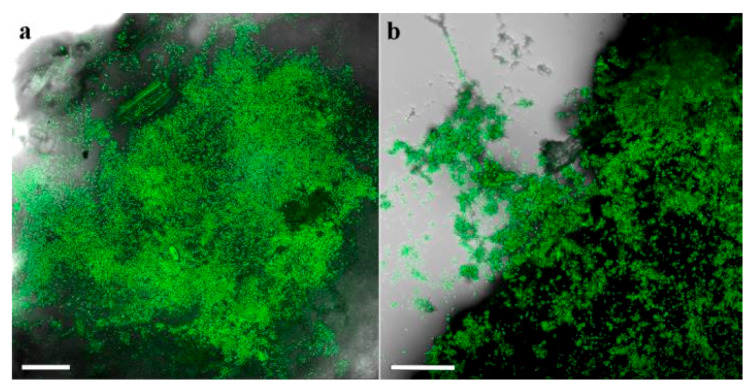
Plant tissue and frass samples from galleries sampled 6.5 months after soil application of EPF: (**a**) Mb7-GFP; (**b**) GHA-GFP. Scale bar = 0.1 mm.

The identity of the fungi on the larval cadavers and frass, and in the galleries found inside the plants was confirmed by detection of the respective GFP transformants under a fluorescent stereomicroscope (Figure 6 and Figure 7).

### 3.4. Survival and Infectivity of the Fungus over Time in Potted Rootstocks

**Figure 8:**
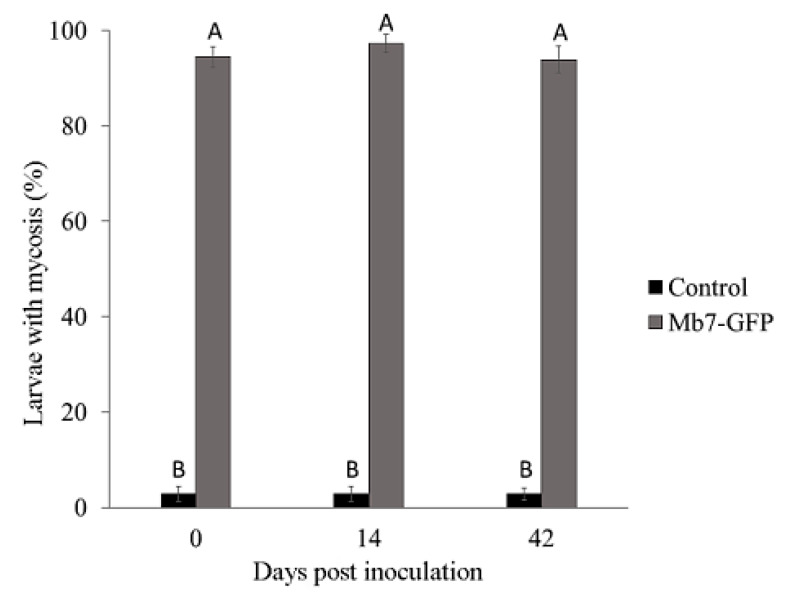
Rate of mycosis in *G. mellonella* larvae exposed to soil samples from potted rootstocks at different times after EPF application in 2018 trials. Data of two rootstocks (2729 and GF677) are combined. Values sharing a common letter are not significantly different. Two-factor ANOVA was performed with replications (*p* = 0.0001).

In the 2018 trial, mortality rate of *G. mellonella* in soil from control rootstocks was significantly lower (1%–3%) than that induced by Mb7-GFP from soil premix treatment (93%–97.5%) for all time points (F = 3445.29, DF = 1, *p* = 0.001). No difference was observed for either treatment over time (F = 0.4206, DF = 1, *p* = 0.521) (Figure 8). No significant difference in the rate of larvae exhibiting mycosis was found between sampled soils used for planting 2729 and GF677 (F = 0.31, DF = 1, *p* = 0.581).

**Figure 9:**
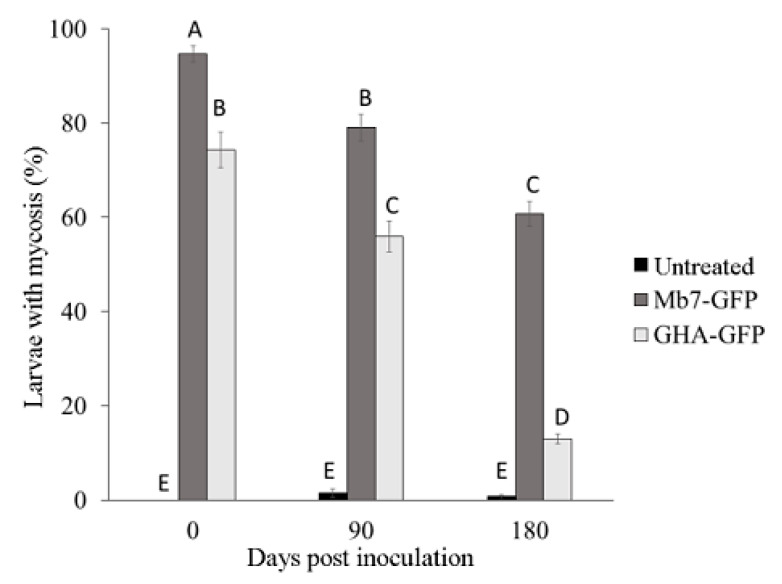
Rate of mycosis in *G. mellonella* larvae exposed to soil samples from potted rootstocks at different times after planting in 2019. Data of two rootstocks (2729 and GF677) are combined. Values sharing a common letter are not significantly different. Two-factor ANOVA was performed with replications (*p =* 0.0001).

In the 2019 trial, mortality rates in control samples (0% for day 1, 2% for day 90, and 1% for day 180) did not differ over time. In the treatments, mortality rates by Mb7-GFP (95% for day 1, 79% for day 90, and 61% for day 180) and by GHA-GFP (74% for day 1, 56% for day 90, and 13% for day 180) differed significantly over time (F = 50.8325, DF = 4, *p* < 0.0001) (Figure 9). There was no difference in the rate of larvae with mycosis between the soil samples of the two rootstocks (F = 1.9058, DF = 1, *p* = 0.1764).

**Figure 10:**
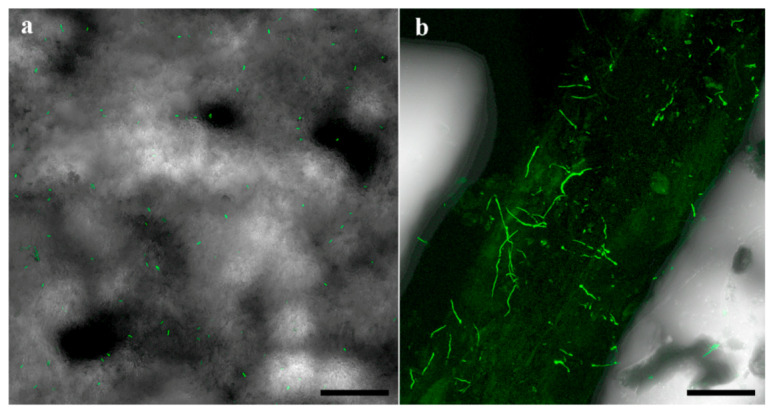
Confocal laser scanning micrographs of Mb7-GFP treated soil (**a**) and root (**b**) samples at 42 days after planting. Scale bar = 0.1 mm.

In the 2018 and 2019 trials, survival of the applied fungi was detected in the soil and around the roots at all time points after planting. In the 2018 trial, Mb7-GFP conidia were detected in the soil (Figure 10a) and germinating conidia were observed on the root surface of GF677 (Figure 10b).

## 4. Discussion

The management of many wood-boring insects in the field is hindered by the long period of their life cycle spent protected under tree bark. The adults, eggs, and newly hatched larvae of these species are the stages exposed to natural enemies and control measures. Unfortunately, no efficient natural enemies of *C. tenebrionis* are known, apart from commercial entomopathogenic nematode products. Mainly, adult control is made by multiple applications of non-specific plant-protection products throughout the growing seasons. Vice versa, sprays targeting eggs and neonates do not prevent plant infestations. However, EPF could be applied in the soil before adult oviposition in order to contrast newly hatched larvae. As the latter have to crawl through the soil to reach the plant, conidia may adhere on their cuticle, thereby facilitating larval infection in the soil and further developing mycosis in the plant.

In this study, pathogenicity of Mb7 and GHA isolates to neonate larvae of *C. tenebrionis* was demonstrated, and the results depended on the method and dose of the EPF application. A dose of 10^8^ Mb7 conidia/g soil in experimental microcosms completely prevented the twigs’ infestation and initial boring. This suggests that early infection of the larvae stopped their activity quite rapidly, preventing them from reaching the cortex or strongly limiting their contact with it. In the potted rootstock trial, a dose of 1.6 × 10^5^ of Mb7 and 1.3 × 10^5^ GHA conidia/cm^3^ soil, applied about 4 months before release of the neonates, reduced rootstock infestation by up to half that assessed in control plants. Larval colonization rates were reduced 7.4-fold in 2729 rootstocks treated with both fungi, but only Mb7 completely prevented colonization in GF677 rootstocks. These data are consistent with previous studies even though the studies differ in the experimental models. In a previous study, *M. anisopliae* isolates induced higher larval mortality than *B. bassiana* isolates when applied by dipping neonates into a suspension of 1 × 10^8^ conidia/mL [16]. A further study showed a positive effect of a spore suspension of *M. anisopliae* isolate EAMa 01/58-Su spread on the soil surface of young potted seedlings of cherry plum, upon examination of the roots 21 days after larval release and 23 days after fungal treatment [17]. The experimental model of the current study in potted plants more closely resembles real-life crop conditions. The exact inoculum of the EPF applied to the premix soil was not assessed from its preparation until larval release. This issue needs to be investigated under different environmental conditions (temperature, soil water content, soil composition, soil microbiome influence), and this was beyond the aim of the current work. Cadavers found in the rootstocks, some of which were protected in the galleries, were confirmed to be infected by the applied fungi but mycosis rates depended on the type of the rootstock. Since the potted rootstocks were examined 2 months after the release of the neonates, it is feasible that most larvae, especially the youngest ones, could not be found and recovered to assess fungus-related mortality, having already decayed. The length of the galleries bored by the larvae might better support the efficiency of the application. These were significantly shorter in all EPF-treated plants compared to the controls, suggesting infection and death of the larvae inside the roots. This interpretation is supported by the detection of fungal mycelia and conidia on frass left by larvae inside the galleries in the roots. This is in accordance with Marannino et al. [16,17], who detected *C. tenebrionis* larvae in the galleries exhibiting mycosis after only 10 and 21 days since larval infection. We also suggest that mycosis in the galleries is promoted by the obvious high humidity prevailing in the microclimate surrounding the boring larvae.

Concerning the mode of application, a grain of rice colonized by Mb7, herein termed fungal granule, mixed into the soil used for planting (premix), was more effective than topsoil spreading of the same EPF before the release of neonates, as demonstrated in 2018 trial. Based on this result, the 2019 trial was carried out by releasing neonate larvae 4.5 months after premix preparation with Mb7 or GHA. These larvae were efficiently controlled, indicating that the amount of conidia in the soil persisted long enough to infect larvae over the period of the trial. Usually, when protecting plants from root-feeding insects using EPF, the focus is on applying large amounts of inoculum to increase the fungal population throughout the bulk soil. However, the effectiveness of EPF for pest control depends in part on the persistence of the applied inoculum in the field [32,33,34]. In our study, each gram of sporulated rice consisted of 1.5–2.5 × 10^8^ conidia and the premix used for planting had 1.3–1.6 × 10^5^ conidia/cm^3^ soil. A similar granular formulation of *M. brunneum* F52 added to potting media at a rate of 0.3 kg/m^3^ was found to persist for two growing seasons, with a decline during the first 7 weeks followed by stabilization until 68 weeks, causing 50%–60% mortality of black vine weevil at that time [29]. Our study indicated that both fungi remained viable for long time as conidia. On one occasion, Mb7 was observed germinating around the root surface. Conidia were observed around root surface at 42 days after its application and high infection rates remained for the first three months after application, according to *Galleria* assay. The importance of plant and rhizosphere associations for persistence over time has been documented for *M. anisopliae* [35] and suggests the involvement of the rhizosphere as a fungal community promoter and potential reservoir for EPF. Rhizospheric and endophytic associations of both *Metarhizium* spp. and *Beauveria* spp. are known to inhibit the growth and reproduction of a wide range of herbivores [36,37,38]. These results suggest the practical possibility of mixing EPF at low rates into soil around the plant trunk that has been ploughed to a dozen centimeters in depth. In order to optimize EPF action, its application could be done possibly about one month before the beetle starts its oviposition. This application timing could favor fungal establishment in the soil before larvae start hatching and cover the long-oviposition time of this species lasting about three months. The lower infestation of untreated GF677 plants is in agreement with the findings of Mendel et al. [5], but not with those of Mulas [39]. Furthermore, larvae reared on artificial substrate that included cortex flour of GF677 [40] did not show reduced growth, in contrast to cortex flour from other rootstocks. The different experimental designs in these studies do not allow a full comparison. However, it can be assumed that GF677 is less attractive and offers a certain resistance to larval penetration, suggesting an antixenotic effect (current results and [5]), whereas the larvae can grow efficiently inside the roots for longer periods of time [39,40].

## 5. Conclusions

The present research addressed questions concerning EPF formulation, delivery mode, and persistence. We demonstrated that *Capnodis* larvae are susceptible to the Mb7 strain of *M. brunneum* and to the commercial GHA strain of *B. bassiana* under laboratory and semiartificial conditions. The mixing of fungal granules (EPF-rice grains) with soil supported survival of the fungus in the rootstock soil over a long period, increasing the chances for fungal infection of the target insect’s larvae. The study also determined the mechanism by which fungal infection occurs and showed that some of the larvae die in the soil and some of them acquire the conidia on their cuticle, infest the plant, and die from mycosis later within the galleries that they have begun to bore. In nature, *C. tenebrionis* lay eggs in the ground at about 7 mm depth (average length of the ovipositor). The lower efficacy of topsoil application method might be related to this egg-laying behavior as well as to the drying of soil surface, which might affect fungi development. The fact that neonate larvae must crawl through the soil to reach plants probably facilitates the initiation of infection by promoting the exposure of a greater proportion of the cuticle surface to the soil inoculum. The EPF-application method tested here to prevent root colonization by neonates needs to be validated in field trials to assess the preventative protection rates for stone-fruit trees. Field trials should also examine the persistence of the fungal granule formulation, and the influence of abiotic and agronomic factors on EPF efficacy and persistence throughout the oviposition period of *C. tenebrionis*.

## Figures and Tables

**Table 1:** Summary of trials carried out in this study.

	Trials	Treatments	Trial Conditions
1	Evaluation of larval neonates’ susceptibility to Mb7-GFP in microcosms in the laboratory	Neonates released on: (a) soil treated with 5 × 10^6^ conidia/g soil (b) untreated soil	20 apricot twigs per treatment
2	Assessment of the relationship between Mb7-GFP dose and mortality of larval neonates in microcosms in the laboratory	Neonates released on: (a) soil treated with 10^5^ conidia/g soil (b) soil treated with 10^6^ conidia/g soil (c) soil treated with 10^8^ conidia/g soil (d) untreated soil	20 apricot twigs per treatment
3	Evaluation of the larval neonates’ susceptibility to Mb7-GFP on potted rootstocks (trial 2018)	Neonates released on: (a) premix soil incubated with 3 g (fungal granules) (7.5 × 10^8^ conidia)/L potting media ^1^ (b) EPF-free soil covered with 5 g fungal granules (1.25 × 10^9^ conidia) per pot (c) untreated soil	(a) 12 plants of GF677, four per treatment (b) 11 plants of 2729, four for untreated soil and three for each treatment
4	Evaluation of the larval neonates’ susceptibility to Mb7-GFP and GHA-GFP on potted rootstocks (trial 2019)	Neonates released on: (a) premix soil incubated with 3 g (fungal granules) (7.5 × 10^8^ conidia)/L potting media ^1^ (b) premix soil incubated with 4 g (fungal granules) (6 × 10^8^ conidia)/L potting media ^1^ (c) untreated soil	(a) Six plants of GF677 and six plants of 2729 (b) Six plants of GF677 and six plants of 2729 (c) Eight plants of GF677 and eight plants of 2729

^1^ Calculated values of Mb7-GFP: 1.6 × 10^5^ conidia/cm^3^ soil and GHA-GFP: 1.3 × 10^5^ conidia/cm^3^ soil.

**Table 2:** Infestation, colonization, and mycosis rates of *C. tenebrionis* larvae in untreated soil (control) and Mb7-GFP treatment (5 × 10^6^ conidia/g soil).

Treatments	Infestation Rate (% ± SE)	Colonization Rate (% ± SE)	Mycosis Rate (% ± SE)
Control (untreated soil)	100.0 ± 0.0 A	86.0 ± 14.0 A	0 ± 0 A
Mb7-GFP treatment	78.0 ± 14.1 A	20.0 ± 5.7 B	60.0 ± 22.4 B

Means in each column followed by different letters differ significantly by Student’s *t*-test. Infestation rate: comparison to 100% by confidence interval, F = 3.79, DF = 1, *p* = 0.123. Colonization rate: F = 15.76, DF = 1, *p* < 0.01. Mycosis rate: comparison to 0% by confidence interval, F = 20.6, DF = 1, *p* = 0.01.

**Table 3:** Infestation, colonization, and dead individual rates of *C. tenebrionis* larvae in untreated soil (control) and Mb7-GFP treatments with increasing doses of conidia.

Treatments	Infestation Rate (% ± SE)	Colonization Rate (% ± SE)	Rate of Dead Neonates on Soil Surface (% ± SE)
Control (untreated soil)	100 ± 0.0 A	81.3 ± 3.9 A	0 ± 0 A
10^5^ conidia/g soil	76 ± 7.5 B	30.0 ± 1.7 B	10 ± 5.2 A
10^6^ conidia/g soil	48 ± 4.9 C	18.0 ± 9.7 B	14 ± 9.8 A
10^8^ conidia/g soil	0 ± 0 D	0 ± 0 C	10 ± 3.2 A

Means in each column followed by different letters differ significantly. Infestation rate comparison to 100% by confidence interval. Rate of neonates on the soil surface and colonization rate comparison to 0% by confidence interval. Bonferroni *p* = 0.05 two-sample *t*-test for means with non-zero standard deviations.

**Table 4:** Trial 2018: infestation, colonization, and gumming rates of *C. tenebrionis* larvae on potted rootstocks treated with Mb7-GFP premix, topsoil application, or untreated soil (control).

Treatments	Infestation Rate (% ± SE)	Plant Colonization Rate (% ± SE)	Gumming Rate (% ± SE)
Control (untreated soil)	75.0 ± 16.4	37.5 ± 18.3	37.5 ± 18.3
Mb7-GFP premix in soil	14.3 ± 14.3	14.3 ± 14.3	14.3 ± 14.3
Mb7-GFP topical application	42.9 ± 20.2	42.9 ± 20.2	28.6 ± 18.4

**Table 5:** Trial 2019: effects of *C. tenebrionis* larvae on potted rootstocks of GF677 and 2729 treated with Mb7-GFP (3 g fungal granules (2.5 × 10^8^ conidia)/L soil) and GHA-GFP (4 g fungal granules (1.5 × 10^8^ conidia)/L soil) premix (average ± SE).

Treatments	Rootstocks	Infestation Rate (%) ^1^	Larval Colonization Rate (% ± SE)	Dead Larva Rate (% ± SE)	Mycosis Rate (% ± SE)	Gumming Rate (%) ^2^	Gallery Length (cm ± SE)
Control	GF677	62.5	6.3 ± 1.8 *α*	1.3 ± 0.8 *α*	0 ± 0 *A*	62.5	23.6 ± 5.9 *α*
	2729	100.0	36.9 ± 4.3 A	3.1 ± 1.6 A	0 ± 0 A	100.0	45.0 ± 3.3 A
Mb7-GFP	GF677	33.3	0.0 ± 0.0 *b*	0.0 ± 0.0 *α*	0 ± 0 *A*	16.7	NA
	2729	50.0	5.0 ± 1.8 B	3.3 ± 1.7 A	3.3 ± 1.7 B	33.3	14.1 ± 4.9 B
GHA-GFP	GF677	33.3	5.0 ± 1.8 *α*	2.5 ± 1.1 *α*	1.7 ± 1.0 *A*	50.0	26.6 ± 11.8 *a*
	2729	50.0	5.0 ± 2.2 B	3.3 ± 1.0 A	2.5 ± 1.1 B	33.3	24.0 ± 11.0 B

Means in each column followed by different letters differ significantly (Greek and italic letter for the comparison among GF677; Latin and plane letter for the comparison among 2729). Comparison to 0% or 100% values was by confidence interval. Bonferroni *p* = 0.05 two-sample *t*-test for means with non-zero standard deviations. Infestation and gumming rate were binomially calculated and were compared by nominal logistic regression: ^1^ Treatment: Chi-Square = 9, DF = 2, *p* = 0.011. Rootstock: Chi-Square = 4.5, DF = 1, *p* = 0.0338. ^2^ Treatment: Chi-Square = 12.78, DF = 2, *p* = 0.0017. Rootstock: Chi-Square = 2.5, DF = 1, *p* = 0.1.
